# 

**Published:** 1913-06-28

**Authors:** 


### The Ammonia Coefficient in Pregnancy.

A great deal of research has lately been carried out along the lines of estimations of the nitrogen output of the body in cases of kidney disease. Especial importance, for instance, is attached by some workers to an increase in the proportion of nitrogen excreted in the urine as ammonia to that excreted in other combinations. When this proportion rises beyond a certain figure, it has been held that certain inferences regarding the state of the kidneys are justified. Whether this rule is as absolute as some believe is doubtful. Professor Hotaling, of Syracuse, New York, has been investigating the "nitrogen coefficient" in a series of sixty cases of pregnancy. His conclusions are as follows: In normal cases, with the same diet and the same amount of exercise, and with absence of toxic symptoms, the ammonia percentage remains normal. But in a great majority of such patients there is some nitrogen retention in the later months of pregnancy. It is impossible to determine the clinical condition of the patient from nitrogen analyses alone, but a low ammonia coefficient is of good diagnostic and prognostic import. There is a marked increase of the ammonia nitrogen in cases with toxic symptoms, and a high coefficient indicates the presence of a grave toxæmia, even when clinical symptoms have not yet set in. In nephritic toxæmia, as opposed to that of pre-eclampsia and eclampsia, the ratio is not particularly high, and this is useful in distinguishing between these conditions, but should not be relied on exclusively. All the available physical signs and symptoms must be taken into consideration in making a diagnosis and in deciding on treatment.

### PRESIDENT POINCARE AND THE FRENCH HOSPITAL.

Dr. James Campbell McClure, clinical assistant to the West End Hospital for Nervous Diseases and physician to the Margaret Street Hospital for Consumption, has been appointed physician to the French Hospital, and it was arranged that he should meet President Poincaré at the French Embassy on Tuesday evening.

### UNIQUE GIFT TO GREENWICH HOSPITAL.

Mr. Henry Cooper Henderson, of Ramsgate, has directed his trustees to offer to the trustees of the Greenwich Hospital two curious coloured prints of naval uniforms by Serres published in 1777, and on the death of the last survivor of the residuary legatees he left the ultimate residue of his estate in equal shares to the Royal Society for the Prevention of Cruelty to Animals, the Hospital for Sick Children, Great Ormond Street, W.C., the Animals' Hospital and Institute, 75 Kinnerton Street, London, S.W., the Convalescent Hospital, Walton-on-Thames, and the General Lying-in Hospital, York Road, Lambeth.

### BEQUESTS FOR ANTI-VIVISECTION.

Miss Annie Goff, of Norwood, has left £50 to the Humanitarian League for the Animal Defence Department; £200 to the British Union for the Abolition of Vivisection, provided that at the time of her death it still adheres to the policy of total prohibition of vivisection; £200 to the London and Provincial Anti-Vivisection Society; £100 to Mrs. William Gordon, of Chiswick, for her work on behalf of homeless cats; £400 to the Personal Rights Association (32 Charing Cross), if Mr. J. H. Levy is actively engaged in the work; £100 to the Social Purity Alliance; £1,000 to the Homes and Refuges for Homeless and Destitute Children; £700 and her grand piano to the Royal Normal College for the Blind at Norwood. The ultimate residue of her estate she left as to one moiety to the London and Provincial Anti-Vivisection Society and the British Union for the Abolition of Vivisection, and the other moiety to Our Dumb Friends' League and the Humanitarian League, to be applied for work for animals, and, if possible, for anti-vivisection work.

### Buildings Contemplated.

Fulwood.—The Preston Guardians have resolved to erect a nurses' home for the workhouse infirmary at a cost of about £4,000.

Leicester.—The Town Council has authorised the visiting committee of the mental hospital to apply to the Secretary of State and the Local Government Board for sanction to additional loan of £3,869 for additions to the hospital.

Marple.—A scheme for erecting a sanatorium at an estimated cost of £15,000 is under consideration by the Salford Town Council; and the purchase of about fifty-two acres of land at Rose Hill, Marple, as a site, for the sum of £2,090, has been decided upon.

Montgomery.—The County Council has adopted a scheme for a new mental hospital, estimated to cost £57,000. The architects are Messrs. Hine and Pegg.

Thirsk.—The Guardians have adopted plans for the conversion of the children's block into an infirmary at a cost estimated at £500.

Vaud, Switzerland.—It is announced that official sanction has been obtained for an expenditure of 1,270,000 fr. (£50,500) for building a hospital for women and children in Vaud.

Wrexham.—The Guardians have under their consideration plans and an estimate of £600 for additions to the workhouse.

### Gifts and Bequests.

The Chelsea Hospital for Women has received £100 from Mrs. Almeric Paget.

The Royal Sussex County Hospital, Brighton, has received an anonymous gift of £50.

The Chelsea Hospital for Women has received from the *Daily Express* Radium Fund a contribution of £250 for the purchase of radium.

A subscription of £10 has been received from Mr. Thomas H. Mann and a donation of £25 from Mrs. Barnes for the appeal on behalf of the *Dreadnought* Hospital, Greenwich.

The late Miss Mary Scholes has bequeathed £100 to the Manchester Children's Hospital, and a legacy of £100 has been received from the executors of the late Mr. William Lees, of Brooklands.

Miss Emily Josephine Troup, of Kent, has left £500 to the Queen's Hospital for Sick Children, Hackney Road, £300 to the Homes for Little Boys at Farningham, and £500 to the Hospital for Sick Children, Great Ormond Street, W.C.

Mr. Robert Joseph McDermott, of Dublin, has left the residue of his estate to the Roman Catholic Archbishop of Dublin for distribution between St. Michael's Hospital, Kingstown, the Society of St. Vincent de Paul, Dublin, and the Mater Misericordiæ Hospital, Dublin.

Mr. Hugh Speed Heal, house furnisher, has left £1,000 each to the Royal Association in Aid of the Deaf and Dumb, the Charitable and Provident Society for Granting Pensions to the Aged and Infirm Deaf and Dumb, and the Institution for the Instruction of Deaf and Dumb Children at Brighton, if either William Sleight or his son Arthur Matlock Sleight shall at his death be headmaster of the last-named institution; but if not the bequest to be equally divided between the two other charitable institutions mentioned.

